# 

**Published:** 1824-10

**Authors:** 


### MONTHLY CATALOGUE OF MEDICAL BOOKS.

**** It having been hinted to us that gentlemen, sending copies of their Works, prefer having their titles given at length, it is our intention, in future, to comply with this suggestion: but it is to be observed, that no books can be entered on this List except those sent to us for the purpose;—finding, in the lists hitherto transmitted, that the names of works have frequently been given as published, which have not appeared for weeks, or even months after.*

The Medical Student's Guide; comprising—Regulations for the Examination of Students at the Royal College of Surgeons, London, and at Apothecaries' Hall; Terms of the Surgical and Medical Practice of the Hospitals and Dispensaries in London; and of the various Lectures, with the Hours of Attendance; Pay of the Medical Department of the Army and Navy, and of the Honourable East-India Company's Service. Also, other Information interesting to the Medical Student. To which is added, a List of the most approved elementary and standard Medical Works.—18mo. pp. 102. London, 1824.

☞ *The Student will find this little Work of use on his first arrival in London.*

Practical Remarks. Part I. on Acute and Chronic Ophthalmia, Ulcers of the Eye, &c. &c. Part II. on Remittent Fever, viz. Simple and Complicated. By THOMAS O'HALLORAN, M.D. Author of Treatises on the Barcelona and Andalusian Yellow Fever Epidemics.—8vo. pp. 148. London, 1824.

Observations on the History and Treatment of the Ophthalmia accompanying the Secondary Forms of Lues Venerea. Illustrated by Cases. By THOMAS HEWSON, A.B. Member of the Royal College of Surgeons in Ireland; Professor of Materia Medica and Pharmacy to the College; and Surgeon of the Meath Hospital and County of Dublin Infirmary, &c. &c.—8vo. pp. 117. London, 1824.

Medicinisch-Chirurgische Zeitung. Von D. JOHAN NEPOM-EHRHART.—Insprnck, 1824.

Manuale Medicum; or, a Medical Pocket for the Use of Students. Adapted to the last Edition of the Pharmacopœia Londinensis. By H. L. SANDERS, Surgeon.—8vo. London, 1824.

Practical Observations on Hydrophobia; with a Review of the Remedies employed, and Suggestions for a different Treatment of the Disease. By JOHN BOOTH, M.D. formerly a President of the Royal Medical Society of Edinburgh; Member of the Royal College of Physicians in London; and one of the Physicians to the Birmingham General Hospital.—London, 1824.

